# Effective factors in non-compliance with therapeutic orders of specialists in outpatient clinics in Iran: a qualitative study

**DOI:** 10.1186/s12913-019-4229-4

**Published:** 2019-06-24

**Authors:** Soheila Naghavi, Mohammad Hossein Mehrolhassani, Nouzar Nakhaee, Vahid Yazdi-Feyzabadi

**Affiliations:** 1 Msc Student in Health Services Management, Social Determinants of Health Research Center, Institute for Futures Studies in Health, Kerman University of Medical Sciences, Kerman, Iran; 2 PhD in Health Services Management, Medical Informatics Research Center, Institute for Futures Studies in Health, Kerman University of Medical Sciences, Kerman, Iran; 3 PhD in community Medicine, Neuroscience Research Center, Institute of Neuropharmacology, Kerman University of Medical Sciences, Kerman, Iran; 4 PhD in Health Policy, Health Services Management Research Center, Institute for Futures Studies in Health, Kerman University of Medical Sciences, Kerman, Iran

**Keywords:** Therapeutic compliance, Clinic, Specialists, Outpatients

## Abstract

**Background:**

Non-compliance with prescribed treatment is an important cause of preventable mortality and economic burden. Recognition of the factors for non-compliance with the therapeutic orders of specialists from the perspective of patients and health care providers sheds more light on the issue for policymakers and stakeholders. The current study aimed at determining the factors for non-compliance with therapeutic orders in outpatient clinics in Kerman, Iran.

**Methods:**

The current qualitative study was conducted using the phenomenological method and semi-structured interviews with 10 patients, five specialists, and four health care managers and treatment officials in outpatient clinics in Kerman. The interviewees were selected by purposive sampling. The codes extracted from the interviews were transcribed using conventional content analysis to identify the viewpoints. The MAXQDA 10 software was used to analyze the data.

**Results:**

The reasons for non-compliance with specialists’ orders were categorized into five themes including patient-related (patient-centered), disease-related, therapy-related, the healthcare provider related (healthcare system), and socioeconomic factors. Themes were composed of fifteen categories and forty-one sub-categories. The dominant sub-categories extracted from interviews were health literacy and knowledge of the patient, communication and patients’ trust in physicians and direct costs of treatment.

**Conclusion:**

This study identified a wide range of different individual, disease, treatment, health care provider, and socio-economic factors and the interactions between them which may result into non-compliance with therapeutic orders prescribed by specialists. Therefore, specific attention should be paid to integrate the service provision system into the collaborative approach of the patient and his/her family in order to promote the level of compliance with therapy and care in planning and policy-making to improve the health service provision system.

**Electronic supplementary material:**

The online version of this article (10.1186/s12913-019-4229-4) contains supplementary material, which is available to authorized users.

## Background

The goal of any prescribed medical therapy is to achieve the desired outcomes in patients. Despite the best efforts of specialists, patients’ non-compliance is still a major problem that prevents the clinicians to achieve the desired therapeutic outcomes. Hence, since 1970s, therapeutic compliance is considered a major concern in clinical settings due to its widespread nature [1].

The World Health Organization defines the concept of compliance as the accomplishment of some behaviors such as taking prescribed medication, healthy lifestyle, and following the recommendations provided by health caretakers [2]; otherwise, non-compliance exists.

Non-compliance with treatment refers to the non-use or discontinuity of the treatment process and inattention or failure to follow the prescribed treatment by the patient. Not taking medication, failure to modify lifestyle or diets, frequent changes in the treatment process, and failure to follow or defectively perform the diagnostic tests, imaging, radiography, etc., are some examples of non-compliance with therapy [1, 3].

According to results of relevant research in Iran, 30% of patients with tuberculosis were non-compliance with treatment regimen [4]. the patients with hypertension regimen compliance was poor in 40% [5]. Another studies also showed among the patients undergoing hemodialysis, the rate of compliance with medication, diet, fluid restrictions, and hemodialysis program was 56.3, 78.9, 70.4, and 78.9%, respectively [6]. Furthermore, results of another study conducted in Iran showed compliance rate was reported to be 62.8–86.3% for oral hypoglycemic medications and 38.8–60.0% for cardiovascular medicines [7]. A study conducted on diabetes patients in Iran showed that 59.4% of patients had poor medication adherence [8].

From the perspective of healthcare providers, non-compliance with therapy is an important clinical issue due to two factors: first, it has significant impacts on clinical and therapeutic outcomes and leads to disease progression, increased visits to outpatient clinics, re-admission, and hospitalization. When patients do not follow the therapeutic plan, the effects of the plan are not realized, which may leads to treatment failure and life-threatening reactions [9]. Non-compliance with therapy can adversely affect patients with diabetes, epilepsy, AIDS, asthma, and high blood pressure, as well as the ones undergoing transplantation [2]. Second, it imposes further financial burden to the community by increasing the cost of treatment and hospitalization [10, 11]. In addition to direct financial impacts, due to reduced productivity, even regardless of negative impacts on patients’ quality of life, it has unwanted expenditures such as increased the time of staying at home, reduced income, and increased traveling and expenditures. In addition, due to patients’ non-compliance with therapy and their failure to report, diagnosis may become difficult for physicians; it adds to the complexity of treatment and results in waste of resources in the healthcare system [12].

Many factors are involved in patients’ non-compliance with therapy. It is vital for the treatment team to be aware of the status of such factors in order to reduce the severity of disease complications in an effective manner [13]. The necessity of this study was qualitative research can help explain the effective factors and identify the factors on non-compliance with therapeutic orders. There is not enough qualitative evidence in this area. The negative effects of non-compliance with therapy should be minimized in order to achieve the desired clinical and economic outcomes. Previous studies mainly focused on particular diseases, especially in hospital environments [3, 6, 14, 15], and there is insufficient evidence of non-compliance with therapy in outpatient environments, especially in Iran. In addition, non-compliance with therapy is a context-sensitive issue that can be different and diverse from one setting to another. It can also be influenced both by supply and demand policies for health care services. Therefore, regardless of the type of disease, the current study aimed at identifying the factors affecting non-compliance with therapy in outpatient clinics in Kerman, one of the largest cities in Iran.

## Methods

The current qualitative study employed the phenomenological method. Phenomenology is a systematic and subjective method used to describe the individuals’ experiences and understand their meanings [16]. The researchers believed that there are some understandable and searchable essences in the phenomena and individuals’ experiences and therefore, they examined the subjective phenomena that have fundamental essences of reality [17].

The study population consisted of outpatients, specialists, and managers of clinics. In this study, the purposive sampling method has been used. In this way, patients who were referred to outpatient clinics and also specialists and clinical managers with at least five years of experience in outpatient settings from diverse specialties with research background were selected. First author (i.e. SN) referred to outpatient clinics in Kerman city and those who voluntarily tend to participate in this study were selected. Study aims were described and the written informed consent was obtained before the interview begins. The study sample consisted of 10 patients, five specialists, and four healthcare managers and treatment officials; thus, 19 interviews were conducted. All interviews were conducted by SN and VYF.

Before the interviews were conducted, a quick review of literatures was conducted as an electronic search through valid databases in order to recognize concepts and provide an interview guideline. The interview guideline was set up based on the review of the most important studies; besides, an additional question, which was necessary to identify each additional factor, was added. The questions included in the interview guide is presented in the appendix, Additional file 1.

Afterward, semi-structured interviews were conducted with the participants on the specifically reviewed subject. After explaining the concept of non-compliance with therapy and the goal of the study to the participants, the researcher conducted the interviews by asking the prepared questions and attempted to pull out their experiences with no specific bias.

Interviews were conducted at the appointed time and place agreed upon by the participants. In order to conduct interviews with doctors and managers, an appointment was made, and the interviews were conducted when they had no patients or at their resting time. Interviews with patients were conducted in the service provision setting. All interviews were audio-recorded and transcribed verbatim. The average time of interviews with specialists and patients was 35 and 20 min, respectively. The minimum and maximum time of the interviews was 10 and 60 min, respectively. The interviews continued until no new reasons for non-compliance with therapy emerged and data were saturated.

Each interview was immediately transcribed verbatim, transferred into the software, and used as a guideline to conduct the next interviews. In order to accomplish this step, the transcribed interviews were transferred into the MAXQDA 10 software and data were coded. Conventional content analysis was employed to code the interviews. Content analysis is a research method used to objectively and regularly describe the obvious content of communication messages [18]. The conventional content analysis is used in any research plans aiming to describe a phenomenon. This method helps to create knowledge, new ideas, fact presentation, and practical guidance for performance through obtaining variable and stable outcomes from textual data [19].

In order to accomplish content analysis, the interviews were independently coded by SN and MHM and any uncertainties and disagreements were discussed with participation of the first author (i.e. VYF) until consensus reached. The transcriptions were reviewed several times to obtain a complete understanding; then, the analysis was performed based on the perception of transcriptions. Some codes were assigned to keywords and phrases that reflected participants’ opinions. In the next step, the codes were compared and in the case of discrepancy, some discussions were made to reach consensus. The codes were reviewed several times in meetings, and were classified based on the similarities and differences of contents. According to the quality of the relationship between the contents of texts, the codes were combined and organized, and the main topics that described the codes were extracted. In the next step, the codes and concepts were defined. In the end, a summary of the definitions and categorized concepts were provided to be reviewed by the researcher. Then, the classification was completed and finalized. We also run and checked all the stages included in a qualitative study according to the important items proposed in the checklist named as criteria for reporting qualitative research [20].

The four criteria proposed by Guba and Lincoln for trustworthiness including credibility, transferability, dependability, and confirmability were used to ensure the validity, accuracy, and stability of the qualitative data [21]. For the credibility of data, sampling was performed with a maximum variety of specialties. Enough time was allocated for each interview; To enhance reflexivity [22], field notes during conducting the interviews with patients and specialists were taken in the diverse steps of the research process and discussed with members of the research team when necessary. Participants were informed that participation was entirely voluntary and they could withdraw at any time. Respondent names were removed during analysis and other identifiers were replaced in the quotations used [22]; the codes extracted from the interviews were given to the participants to be approved. In order to enhance transferability, experienced managers and specialists with different kinds of specialties were interviewed, and the interviewed patients did not have similar diseases. Most of the study results were confirmed in other studies. It was tried to completely explain all study steps, the field of work, and environment for the readers. To ensure the dependability criterion, interviews were coded separately by two individuals, in addition to the research team, and reviewed several times; the study protocol was provided to professors experienced in qualitative research. According to the three previous criteria, it can be claimed that confirmability was observed automatically.

we employed triangulation in sampling. We used main different stakeholders for sampling, including healthcare and treatment officials, specialists, and patients who are directly related to the phenomenon of non-compliance in therapeutic orders.

The current study was approved by the ethics committee of Kerman University of Medical Sciences with ID number IR.KMU.REC.1397.040. No personal information is reported here, and all names are pseudonyms (i.e. P = Patient; M: Manager; S: Specialist). Notes were taken during interviews and if participants agreed, they were audio-taped.

## Results

Among the healthcare specialists and managers, 45% were male and 55% female; 67% were faculty members and 33% non-faculty members; 56% were specialists and 44% healthcare services managers or treatment officials. Among the participating patients, 40% were male and 60% female and their education level ranged illiterate to Master of Science (Table 1). A summary of the demographic characteristics of the participants including patients and specialists and managers is presented in Table 1.

**Table 1:** A summary of the demographic characteristics related to patients (*n* = 10), specialists, and healthcare managers (*n* = 9)

Participant group	Variable	Frequency (%)
Patient	Gender	
	Male	4 (40.0)
	Female	6 (60.0)
	Education level	
	Illiterate	1 (10.0)
	Diploma and lower	5 (50.0)
	Academic/university	4 (40.0)
Healthcare manager/specialist	Gender	
	Male	4 (44.5)
	Female	5 (55.5)
	Faculty members/ Non-Faculty members	
	Faculty members	6 (66.7)
	Non-Faculty members	3 (33.3)
	Scope of activity	
	Clinical specialists	5 (55.5)
	Healthcare services managers or treatment officials	4 (44.5)

**Fig. 1:**
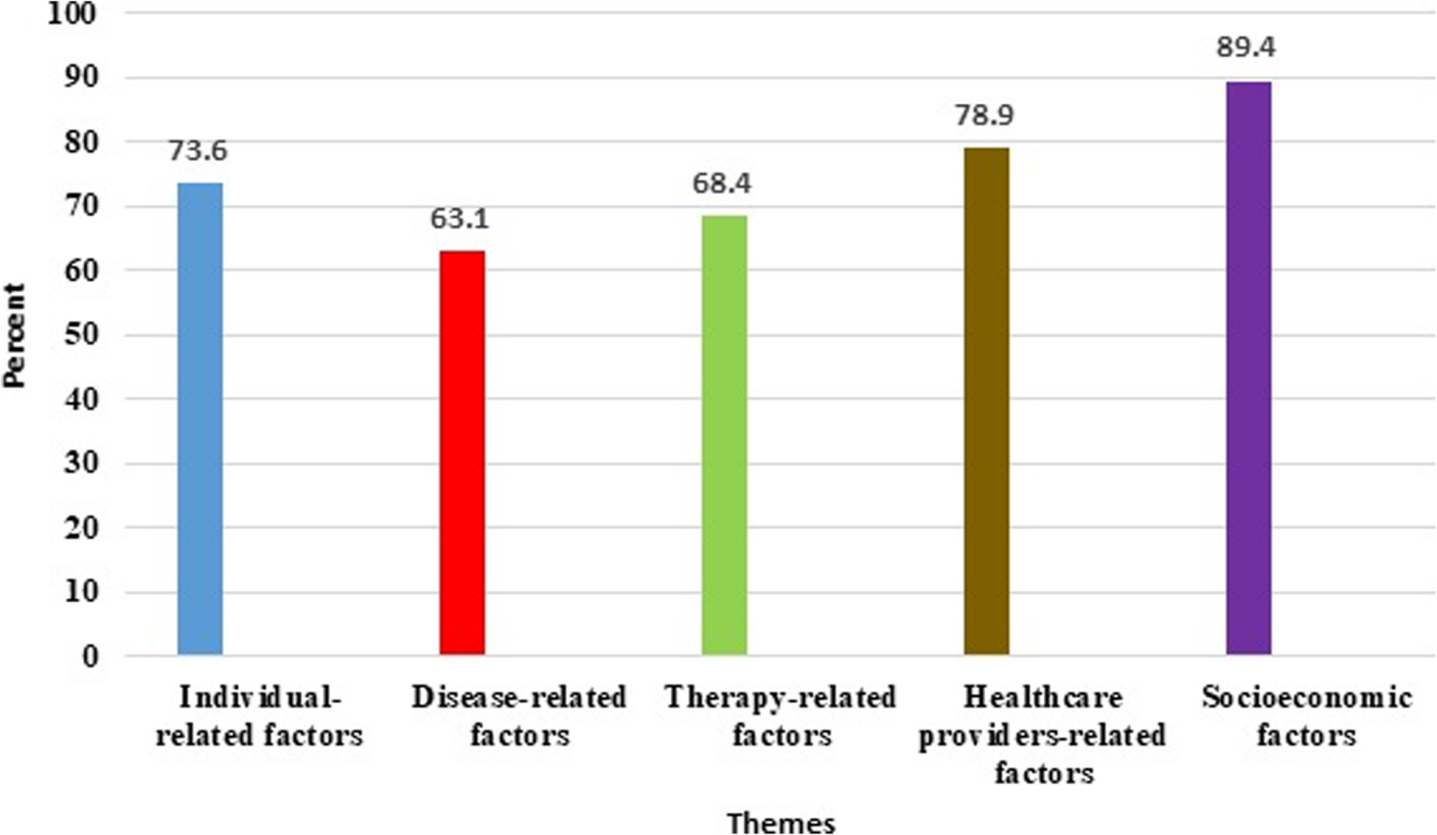
Frequency of the themes extracted from the interviews

The factors identified from the analysis of interviews with specialists and patients were classified into five themes, fifteen categories, and forty-one subcategories. The themes extracted were including individual-related (patient-centered), disease-related, therapy-related, the healthcare providers-related (healthcare system), and socioeconomic factors (Table 2). Furthermore, the frequency of the themes obtained from the interviews is presented in Fig. 1.

**Table 2:** The themes, categories, subcategories and codes related to the non-compliance of the therapeutic orders prescribed by specialists

Theme	Category	Subcategory	Code
Individual-related (patient-centered) factors	Demographic factors	Age	
		Gender	
		Educational level	
		Marital Status	
		Job type	
		Ethnicity	
	Cognitive factors (beliefs)	Health literacy	
		Patient knowledge	
		Health importance degree for the individual and health motivations	
		Individual’s recognition and perception from nature	
		Previous experience	
		Religious beliefs’ perception and believing	believe in specialisms
			cultural features and values
			Ethnic-religious beliefs
			Folk perspectives and non-professional interventions
			Individual’s perception, expectations and judgment from treatment period
			Medications effectiveness perception
			Mental stereotypes
		Reasoning power and decision- making attitudes	
		Forgetfulness	
	Psychological factors (negative attitudes toward treatment)	Fear of disclosing information (stigmatization)	
		Obsession	
		Depression and weakness	
		Anxiety	
		Fear and denial	
	Behavioral factors	Behavioral habits and individual’s ability to change them/behavioral habits and lifestyle	
Disease-related factors	Disease type		
	Disease stage and severity		
	comorbidities (disease complexity and having chronic diseases)/ Underlying diseases		
Therapy-related factors	Route of administration		
	Treatment complexity		
	Duration of the treatment period		
	Medication side effects		
The healthcare provider-related (healthcare system) factors	Individual attributes	Ethics and behavior (professional ethics)	
		Background and experience	
		Therapist’s literacy	
		Fame	
		communication and patients’ trust in physicians	
		The extent of doctor’s recognition from patients and doctor’s decision-making attitude (prescribing a lot or few amounts of medications)	
		Power enforcing attitude (therapist to patient)	
		Professional principles	Enough time (therapist for the patient)
			Regular attendance (therapist)
			Guidelines following
	Service provision system characteristics	Ownership and nature	
		Service provision system structure	Referral system
			Interaction and communication mechanisms between therapist and patient (participation, decision-making and interaction attitudes)
			Intervention of specialties and lack of patient guidance system
			Standards and protocols
		Payment system	Payment mechanism (type)
			Level of payment (price)
			Insurance coverage/basic and supplementary
		Access	Waiting time
			Physical facilities
			Geographical access
			Medication access (medication rareness)
			Informational infrastructures
			Equipment and technology
		Legal Guarantees	Lack of supervision
			Doctor’s misconduct/the claim of doctor’s misconduct
			Corruption in service provision
		The systematic interactions between traditional and modern medicine	Convergence between two types of medicine
			Different application of two medicines in the disease treatment
Socioeconomic-related factors	Economic issues	Direct costs of treatment	Possibility of costs payment by household (low income)
		Indirect costs of treatment (transportation, diet)	
	Social issues	Job security (anxiety about job loss)	
		Concerns and environmental stresses (especially in the workplace)	
		Advertising, Media and Cyberspace	
		Pride in treatment	
		Supportive system	Family
			Friends and relatives
			Social groups

### Individual-related (patient-centered) factors

Individual factors of patients were classified into four categories including demographic, cognitive (belief), psychological (negative attitudes toward treatment), and behavioral factors.

#### Demographic factors

The factors identified in this class consisted of six subcategories, including age, gender, education level, marital status, job type, and ethnicity.

With aging, individuals may have vision, hearing, and memory problems affecting how to take their medications (such as swallowing problems, precise identifying of the medications), and they may not be able to meet compliance with therapy. In addition, with aging, due to reduced physical and mental abilities, they need the others to remind and prepare their medications for them. The interviewees mentioned age as a parameter that can be related to non-compliance with therapy.

*“When an individual becomes older, his/her level of hearing is lower, and there is the possibility that doctor’s statements are not heard correctly, and the instructions are not received accurately” (M1).*

*“For some patients, especially with aging, no one may exist in order to prepare their medications for them; necessary therapeutic actions may be interrupted for two or three months or treatment process may be done with delay” (S4).*

*“As people grow older, they become more timid and conservative, and they pay much more attention to the recommendations and instructions than young people” (P8).*

Of the interviewed individuals mentioning gender, most of them stated that males are more prone to non-compliance than females.

*“Males usually show less compliance with therapy than females; therefore, gender can be an effective factor in non-compliance” (S1).*

*“I think females have better compliance compared with males, since they are sensitive even to very minor problems, and they quickly refer to doctors and try to do as the doctors say. However, males are more carefree, and they say that there is not a very important problem, except in the cases that their diseases become very serious; then they pay attention to the doctors’ instructions” (P8).*

In terms of education, interviewees had different opinions. Some of them believed that people with higher levels of education have better compliance, and some of them agreed with its opposite. The patients said:

*“Education can also be effective. I think that individuals with lower levels of education have better compliance; for example, some uneducated people around me believe that they should follow doctor’s instructions, or they should accurately take their medications based on the doctor’s instructions. However, an educated person may think that some of these instructions are not necessary, or the medication dose is excessive, and they may not have complete compliance” (P8).*

*“Individuals with higher levels of education have better compliance as a result of their higher level of knowledge and due to the issue that they are more aware of the importance of doctor’s instructions” (P5).*

People are involved in different work areas in the community. When people become ill, their work area may affect their compliance, as one of the interviewees stated:

*“……how do the doctors themselves take their treatment, either they themselves diagnose their disease or they behave like ordinary people; either they refer to different doctors or since they themselves are doctors, they believe that their own diagnosis is more accurate; whether they follow non-compliance with specialists and how is the compliance status of general practitioners with specialists? Either the specialty and work area of an individual is related to health sector or he/she is working in other areas. What is his/her specialty? ....” (M1).*

The interviewees also mentioned the individual’s job type and its characteristics such as job-related travels and job conditions, as factors that can lead to non-compliance:

*“….a driver may not use his/her prescribed soporific medications due to his/her job” (P7).*

#### Cognitive factors(beliefs)

The individual factors related to cognition consisted of eight subcategories, including health literacy, patient knowledge, health importance degree for the individual and health motivations, individual’s recognition and perception from nature, previous experience, religious beliefs’ perception and believing, reasoning power and decision-making attitudes and forgetfulness.

An individual’s inability to receive, interpret, and perceive the primary information, health instructions, and services that are necessary to make a proper decision can affect non-compliance. A manager and a specialist in community medicine said:

*“….what is the recipient’s health literacy level? Are the doctor’s instructions understandable for the patient? For example, if the doctor says to the patient that take this medication three times a day, is its meaning understandable for the patient in terms of his/her cognition? Some phrases may also be literally understood differently; for example, if the doctor says to the patient to come tomorrow, he/she might think that he/she should come two days later since in his/her culture, in order to say come tomorrow, you should say come “morning”(M1).*

*“People’s low literacy makes them as individuals who are not aware of the importance of following doctors’ instructions” (M2).*

The lack of patients’ information and knowledge about diseases and treatment, lack of their awareness of complications and benefits of therapeutic orders and the consequences of non-compliance were among the factors mentioned in the interviews.

*“Some patients have chronic diseases. They cannot accept their diseases or it is hard for them to accept their diseases. For example, to some extent, they control their blood sugar or pressure and after that they think they become better and it is not necessary to follow doctors’ instructions anymore. Consequently, they refer again to doctors with high blood sugar or pressure” (S4).*

*“The patient does not have enough information about the disease and medications. For example, the patient may not take his/her antibiotics completely, and he/she is not aware of the consequences of taking such medications incompletely. Therefore, due to his/her insufficient knowledge, he/she takes his/her antibiotics only for some days, and after becoming better, no longer takes his/her medications” (S2).*

*“The individuals may not be aware of the consequences that their non-compliance can cause for them” (P2).*

The importance degree that individuals give to themselves is different. In addition, the individuals’ motivation for treatment may be different, and it seems that this issue can affect patients’ non-compliance. Another factor that can affect individuals’ non-compliance is their experiences. If an individual has an unpleasant experience from his/her previous referral to a doctor or treatment process, this can lead to non-compliance in the patient. In this regard, it was stated:

*“The mindset that the patient has from his/her previous referrals to doctors may create this feeling in him/her that income is more important for them than their patients” (P6).*

To believe in specialisms, cultural features and values, ethnic-religious beliefs, folk perspectives, and non-professional interventions, individual’s perception, expectations and judgment from treatment period, medications effectiveness perception, and mental stereotypes may lead to patient’s non-compliance with therapeutic orders.

*“How are an individual’s recognition and perception from nature, life’s philosophy, and its purpose? And how is an individual’s perspective about his/her being against the world? If the patient considers his/her healthfulness as a deposit, his/her therapeutic compliance will increase. If the patient considers his/her healthfulness as a right, he/she may have a lower level of compliance. Therefore, an individual’s recognition about how his/her worldview can affect his/her therapeutic compliance” (M1).*

*“How is the community’s culture, in which the individual lives, towards the specific therapeutic method? For example, a particular treatment may not be accepted by the residents of a specific village or city according to their customs” (P5).*

*“How are the individuals’ religious beliefs and their perspectives about healing and miracle? Do they follow their treatment process along with their beliefs in healing and miracle or they abandon their treatment process because of those beliefs?” (M1).*

*“Many non-professional individuals such as neighbors and family members interfere with medical issues. These people state their experiences for the patient, and there is even a proverb in this regard, which says “Do not visit a doctor, visit a person who had the same disease.” For example, if someone has a kidney stone, the other individuals around the person that had the experience of kidney stone may give him/her guidelines. Most of the time, in such cases, individuals don’t know that the clinical conditions of patients may be different. A therapeutic method for a kidney stone with a diameter greater than 1 cm in the different places of the kidney may not be similar to the therapeutic method of very small kidney gravel” (S3).*

*“People’s attention to the others’ recommendations…. anyone may suggest a doctor whose performance is better than the others, and unfortunately, some people pay much attention to others’ suggestions” (P6).*

*“Some people refer to various doctors, and since they do not become better after a long period of time, they become disappointed and lose their motivations for any further treatments. For example, one of our acquaintances referred to doctors for infertility treatment for eight years, due to not getting desirable outcomes from her treatment during this long period of time, became disappointed and abandoned her treatment process” (P7).*

Some individuals consult with other people when they want to decide on doing doctor’s instructions, and therefore, due to this consultation, the patients may become hesitant in decision-making and following doctor’s instructions. In some cases, the patient may forget the prescribed instructions. The patients said:

*“Sometimes, we may forget the doctor’s instruction, especially about taking medications or when the doctor says to us please refer again at that time. We may remember these issues after a while” (P1).*

*“Sometimes, people say different things to you and you get confused. For example, when you have a disc herniation, the doctor may say to you that you need surgery to become better, but some people who are around you may say to you that surgery cannot be effective and you may get worse. In such cases, you get completely confused.” (P9).*

#### Psychological (negative attitudes toward treatment)

The psychological factors that patients mentioned as factors for therapeutic non-compliance were classified into five subcategories, including fear of disclosing information (stigmatization), obsession, depression and weakness, anxiety, and fear and denial.

The participants stated that the patient might not follow his/her treatment process as a result of stigmatization and fear. In addition, it was stated that obsession in the form of irrational and annoying thoughts or conceptions compel an individual to do an action repeatedly, forcibly, and involuntarily, for example, frequently changing his/her doctors. Depression and weakness, anxiety, and fear are among the other factors that interviewees mentioned as non-compliance factors:

*“An addict that consumes drugs is often afraid of going to the laboratory, since he/she fears that someone may discover his/her addiction….” (P6).*

*“Sometimes, people do not refer to doctors since they are afraid that the outcomes of their therapeutic actions (tests, sonographies, etc.) may indicate a dangerous disease. They say that I am ok now, and if I refer to a doctor, I will become ill” (P6).*

#### Behavioral factors

Habits and the individual’s ability to change them (behavioral habits and lifestyle) were classified in this category.

Participants stated that individuals have different lifestyles. They do not accept some of their behavioral habits such as diet restrictions and smoking avoidance, and they cannot change them easily.

*“Sometimes, an individual is in a party in which all people are eating very delicious food. In such a situation, although the individual knows that he/she should not eat that food, he/she cannot control himself/herself and consequently eats it” (P9).*

### Diseases -related factors

Disease characteristics were classified into three categories, including disease type, disease stage and severity, and comorbidities (disease complexity and having chronic diseases)/ Underlying diseases.

The type of individual’s disease and its symptoms, the disease appearance and manner, the disease stage and severity, and having several diseases simultaneously can affect non-compliance. The specialists said:

*“Non-compliance is different based on the disease severity; either the disease is at early stages and does not have a lot of symptoms or the disease severity is very high and it has a lot of symptoms” (S5).*

*“Maybe, the patient has several diseases simultaneously, and it is necessary for him/her to take a large number of medications for each of his/her disease, which is not possible for him/her” (S4).*

### Therapy-related factors

Therapy-related factors consisted of four categories including the route of administration, treatment complexity, duration of the treatment period, and medication side effects.

The prescribed treatment method may not be compatible with the person, or treatment complexity and the other chronic diseases of an individual in addition to his/her disease can lead to non-compliance. The long-term treatment period of some diseases and the side effects of medications can also lead to non-compliance.

*“Sometimes, we get tired of continuously taking medications, and we do not have any motivations to continue treatment.” (P2).*

*“The side effects of some medications; for example, I referred to a dermatologist, and he prescribed tetracycline for me, and since I have also bowel disease, I could not take this medication” (P6).*

### The healthcare provider-related (healthcare system) factors

The healthcare-provider-related factors were classified into two categories, including the Individual attributes and service provision system characteristics.

#### Individual attributes

The Individual attributes mentioned in the interviews were classified in eight subcategories, including ethics and behavior (professional ethics), background and experience, therapist’s literacy, fame, communication, communication and patients’ trust in physicians, the extent of doctor’s recognition of patients and doctor’s decision-making attitude (prescribing a lot or a few number of medications), power enforcing attitude (therapist to patient), and professional principles.

*“The features and attributes of a doctor in his/her medical performance, the level of his/her accuracy, and his/her literature in relation with patients are important factors. The same medication may have different effects if it is prescribed by doctors with different personal features” (S3).*

*“It happened several times to me that I referred to doctors, and they could not diagnose my disease or misdiagnosed. Therefore, I think that some doctors have insufficient knowledge, and cannot diagnose accurately” (P1).*

*“How is the doctor’s verbal communication, and how does the doctor listen to patient’s talk? For example, my mother refers to a doctor who lets her speak about her disease. Then, my mother says that the doctor was very good, and consequently, she follows his/her instructions more accurately and much better” (P3).*

*“Some doctors do not pay any attention to patients’ opinions. They do as they wish, since they think that only they can understand, while they may prescribe something for the patient that it is hard to follow. But, if they ask patient’s ideas in this regard, it would be much better” (P10).*

*“Sometimes, you call a doctor’s office, and his secretary says “the doctor comes at 11 a.m.” But when you go to the office, the doctor comes at 2 p.m.; or sometimes after waiting for an hour, the secretary may say “please go, the doctor comes at 5 pm” (P2).*

#### Service provision system characteristics

Regarding the service provision system, the interviewees stated six subcategories that could affect the non-compliance. These subcategories were ownership and nature, service provision system structure, payment system, access, legal guarantees, and the interaction of traditional and modern medicine. A specialist in community medicine and a patient said:

*“... If they refer to a government system, other people label them, and thus they refer to a private system” (M2).*

*“Since the specialties are very various, and many of the diseases may relate to several specialists, the patients in such cases cannot decide which specialists they should refer to, and they may refer to several doctors for several times to find the main cause of their disease” (P6).*

The determination of payment method for different services may lead to doctors’ unnecessary prescription for their patients. The level of payment is the price specified for the services, and it is very important. There are different types of insurance, and the number of services covered by each of them is different. Therefore, the type of patient’s insurance can be effective for non-compliance.

*“Incorrect and low tariffs in clinics increase the unnecessary visits and non-compliance” (S5).*

*“When we refer to receive health services, it is very important for us to know that how many of the services are covered by the insurance …” (P5).*

Access was one of the factors that the interviewees stated. The concept of access is related to the opportunity and ability to use services; actually, it means the existence of a set of circumstances in which an individual that needs health services can properly use them. The queue is sometimes so long that the person cannot receive the services, leading to his/her leave. In addition, the lack of physical facilities, geographical access, medication access (medication rareness), information infrastructures, equipment, and technology, each can be a reason for non-compliance.

*“The services access facility; whether the services are available to the individuals or they should spend a lot of time in order to receive the desired services either in terms of waiting time and bustle or passed distance” (P7).*

*“Some medications, especially the ones necessary to treat specific patients cannot be found easily, or they may only be found in some metropolises” (P2).*

*“How is our information system? How are the information infrastructures in our health system? Do we have electronic health records? For example, when we refer to a doctor, and he/she wants to see our radiology result for diagnosis, it would be very useful if he/she can see our previous radiographs during the past 10 years in his/her computer. However, if he/she cannot have access to our previous radiology and ask us to repeat it, then we may say that I cannot do it since I have done it recently, and then, the level of non-compliance will increase. Therefore, information infrastructures can be very effective, especially if there is an information system, which can identify that a patient has referred to several doctors for his/her disease. Moreover, especially, when insurance systems can control the patient’s referrals to doctors, and there is a disease care system to control the patient and if he/she shows inappropriate behavior, then this system can follow up this patient and help him/her to solve his/her problem” (M1).*

Lack of supervision, (the claim of) doctor’s misconduct, and corruption in service provision are among the other system-related factors that can affect patients’ non-compliance.

*“There are some people who are not doctors; but due to lack of supervision or the existing beliefs, some patients refer to them, and they provide some services for those patients. Even, some of these fake doctors, unfortunately, ask the patients to follow the instructions they give, and that the patients should stop all other therapeutic orders and protocols” (S3).*

*“When a patient observes the unethical communications of doctors with specific drugstores, laboratories, etc., he/she may not pay much attention to those doctors’ instructions anymore” (P4).*

The systematic interactions between traditional and modern medicine affect non-compliance; whether these two systems have conflicts with each other or one of them is more efficient from the individuals’ perspective.

*“Individuals’ beliefs toward traditional or chemical medications are different. For example, I refer to a doctor and he/she prescribes medications for me; since I think chemical medications are harmful, I use traditional ones. However, some of our family members believe that chemical medications are more effective for their treatment” (P8).*

### Socioeconomic-related factors

This theme comprised two categories including economic and social issues.

#### Economic issues

The economic issues of non-compliance with doctor’s instructions consisted of two subcategories including direct and indirect costs of treatment.

Low income and inability to pay health costs are among the most important factors mentioned in the interviews. During the disease period, in addition to the main costs of treatment, other indirect costs of treatment such as transportation, residence, diet are imposed on the individual; due to these extra costs that the individual may not be able to pay, he/she, unfortunately, is forced not to follow the doctor’s instructions anymore.

*“Expensive medications and treatments can play an important role. Some people cannot pay the costs of some medications and treatments; therefore, they give up purchasing their medications and following their treatments” (M3).*

*“Sometimes, doctors recommend eating a special kind of fruit or other items for the patient, and in some cases the patient’s financial condition is not very good, and he/she even hardly purchases his/her essential necessities. Consequently, he/she cannot bear the additional costs of doctor’s instructions.” (S1).*

*“Sometimes, the patient’s medication may become over, and at the same time, he/she may not have enough money to buy his/her medication again. Therefore, taking medication stops for that patient” (P10).*

#### Social issues

Social issues consisted of seven subcategories including job security (anxiety about job loss), concerns and environmental stress (especially in the workplace), advertising, media and cyber space, pride in treatment, and support system.

Sometimes, compliance with doctors’ instructions may have inappropriate occupational outcomes for the individual; therefore, the individual prefers non-compliance with them. Some interviewees also mentioned that some people have such complicated social concerns and problems that the issue of health and compliance with doctors’ instructions become unimportant for them.

*“Although the fact of disease’s importance and the issue that the disease is with you until the end of your life were explained to the patients, due to their high level of concerns and engagement, they did not pay attention to completion and follow-up of their treatment” (S4).*

*“The people’s concerns are so great that the issue of medication compliance is unimportant to them…” (M2).*

Due to extensive communications and easy access to information, many people, when referring to doctors to receive prescribed instructions, search the prescriptions on the Internet or adapt them to the information that they receive from media and cyber space; if they find some contradictions between them, they may decide not to follow those instructions.

*“Patients compare the doctors’ instructions with the materials they have read on the Internet, and in this way, they obtain information about the subject” (P3).*

Insufficient support from family, friends, and social groups is another reason for non-compliance.

*“The social support system of the person who needs treatment is important. For example, if the patient is too young, how is his/her parents’ support system for his/her compliance with the disease? And, if the patient is old, how do the individuals who support him/her, for example, his/her children affect the patient’s decision making, and conduct psychological support?” (M1).*

#### Social issues

Social issues consisted of seven subcategories including job security (anxiety about job loss), concerns and environmental stress (especially in the workplace), advertising, media and cyber space, pride in treatment, and support system.

Sometimes, compliance with doctors’ instructions may have inappropriate occupational outcomes for the individual; therefore, the individual prefers non-compliance with them. Some interviewees also mentioned that some people have such complicated social concerns and problems that the issue of health and compliance with doctors’ instructions become unimportant for them.

*“Although the fact of disease’s importance and the issue that the disease is with you until the end of your life were explained to the patients, due to their high level of concerns and engagement, they did not pay attention to completion and follow-up of their treatment” (S4).*

*“The people’s concerns are so great that the issue of medication compliance is unimportant to them…” (M2).*

Due to extensive communications and easy access to information, many people, when referring to doctors to receive prescribed instructions, search the prescriptions on the Internet or adapt them to the information that they receive from media and cyber space; if they find some contradictions between them, they may decide not to follow those instructions.

*“Patients compare the doctors’ instructions with the materials they have read on the Internet, and in this way, they obtain information about the subject” (P3).*

Insufficient support from family, friends, and social groups is another reason for non-compliance.

*“The social support system of the person who needs treatment is important. For example, if the patient is too young, how is his/her parents’ support system for his/her compliance with the disease? And, if the patient is old, how do the individuals who support him/her, for example, his/her children affect the patient’s decision making, and conduct psychological support?” (M1).*

## Discussion

Based on the current study results, five themes were extracted indicating the factors for non-compliance with therapeutic orders. The study results showed that various factors related to individual, disease, therapy, service provider, and social and economic issues affect non-compliance with receiving services in outpatient clinics. Some of these subcategories such as health literacy, patient knowledge, communication and patients’ trust in physicians and direct costs of treatment are among the most important ones mentioned in most interviews.

The overall framework to categorize the themes was consistent with that of a systematic review of studies that examined the factors influencing treatment acceptance from the perspective of patients [1]. Gholamaliei et al., in their study on patients with diabetes found that numerous individual, economic, and social factors can affect patients’ non-compliance. In their study, age, level of education, cost of care and treatment, characteristics of the treatment team and the health system, factors related to the disease and treatment conditions and patient’s beliefs were consistent with those of the current study [8].

One of the themes that the interviewees mentioned as an effective factor of non-compliance with therapy was individual-related factors. Butterworth et al., stated that patients that can read and understand medication labels have better compliance [23]. Sheppard-Law et al., found that people with insufficient health literacy were more likely to show non-compliance [24]. Other studies also pointed to this issue [25–27]. Some studies show non-sufficient knowledge of the patients about the disease, treatment, and its consequences leads to non-compliance, and health providers should present enough information about treatment and disease for them [28–30]. Some patients think that they should only take their medications for a specific period, and they should not take them anymore after the end of that period, while it is possible that they continue their medications [31]; for such factors, patients’ training is very important to increase compliance [32]. When a person becomes ill, if his/her knowledge about the disease and treatment is sufficient, he/she can control the disease and treatment much better. One of the other issues that could be understood from specialists and patients’ talks was that people, at the time of disease and treatment, may encounter interventional and therapeutic suggestions of other people about their disease, or they may start reviewing the conditions and consequences of patients with similar conditions; consequently, increasing patients and their relatives’ knowledge in this regard can be very helpful. In a study conducted by Kripalani et al., people with lower health literacy had better compliance possibly due to the point that people with lower health literacy were more confident in their medication taking, but those with higher health literacy sometimes had unintentional admission [33].

Emotional and psychological aspects were among the other issues that interviewees mentioned as individual factors; in this regard, previous studies also showed that shame, discrimination [34], depression, anxiety [35, 36], fear, and denial [28, 37], were effective in non-compliance.

The disease and therapy-related factors were the other themes that interviewees discussed. Numerous studies also indicated that treatment compliance can be affected by disease type [25, 37], disease stage and severity, and having comorbidities [35, 38]. Moreover, suitable medication prescription that is compatible with the patient [39] and considering medications side effects [28, 40] play important roles.

communication and patients’ trust in physicians were among the main factors related to service providers pointed out by many interviewees for their non-compliance. Not listening to the patients’ talks, not providing adequate explanation by doctors about the disease and justification of patients about the disease, the necessity of following instructions and convincing patients to comply with therapeutic orders and lack of patients’ participation in planning their treatment plan are among the factors that increase non-compliance. Bermejo et al., in their study indicated trust plays key roles in treatment, participation, and encouragement [37]. In addition, the doctor’s relationships with patients maintain their motivation for treatment [41]. Several studies show that the relationship between patients and doctors is one of the strong factors affecting patients’ compliance, and a healthy, trust-based relationship can be useful to reduce non-compliance [42–44]. Boru et al., concluded that poor relationships between health providers and patients lead to non-compliance [34]. Moreover, as another service provider-related reason for patients’ non-compliance (leading to stopping therapeutic orders or changing the doctor) it could be pointed to the lack of accurate diagnosis of doctors and not achieving desirable treatment outcomes described by patients, while clinical specialists claimed that this issue was related to the early judgment of the patients and not completing the treatment period.

Other important factors for non-compliance are social and economic issues. In this regard, the results of the study showed that some of the doctors prescribe medications for patients without paying much attention to their economic conditions. Some people have low incomes and they are not able to buy some services; thus, the high cost of some treatments can be among the factors for non-compliance. Therapeutic costs can be a large part of patients’ living costs, and in lower socioeconomic categories, such costs increase non-compliance [38, 45]. Studies show that economic factors are one of the important determinants of treatment non-compliance [34, 46, 47].

Goodhand et al., in their study on patients with inflammatory bowel found that non-compliance was more frequent in patients with lower socioeconomic status [35]. The results of Rafiee- Vardenjani’s et al. study showed that the economic problems were associated with treatment outcomes in patients undergoing hemodialysis [6], which confirmed the results of the current study. Studies show that compliance is much better in patients supported by their family members, friends, or service providers [35, 36, 48]. It seems that the economic condition has an important role in the treatment continuation, and social supports help individuals to have much better motivations to follow their treatment.

### Study limitations and strengths

This is one of the first studies to our knowledge that a qualitative investigation has been undertaken of compliance with therapeutic orders prescribed by specialists within the setting of outpatient clinics in Iran. Our study also had some limitations. One limitation of this study is related to the generalization of the results considering qualitative nature of the study. This study was not designed to be generalizable, but rather to explore providers and patients’ perspectives on the factors affecting the compliance and noncompliance of the therapeutic orders. Furthermore, although many participants were very exposed to declare their perspectives during interviews, they may still have withheld some of their thoughts about noncompliance of the therapeutic orders prescribed by specialists. Furthermore, the selected patients and providers in this study may not be representative of all patients and providers. Since the current study was related to outpatient clinics in Kerman, and due to the different conditions of the individuals and the system in terms of transferability, the results should be cautiously interpreted for other settings and contexts. Another limitation is that although there is a relatively strong research background related to non-compliance with therapeutic orders, most these studies are disease based and often conducted on hospitalized patients in Iran. Thus, there was no evidence enough using similar methodology about outpatient clinics in Iran which limited the comparisons with local evidence. So it is necessary to be conducted further research to identify the factors affecting the compliance and adherence with prescribed therapeutic orders. Precipitancy, stressful environment and patients’ impatience in outpatient clinics’ interview environment were among the other limitations; however, it was tried to use a quiet environment to interview the patients. Lack of cooperation of some specialists due to their busy was another limitation, which was tried to overcome by conducting the interviews with several referrals and getting an appointment with them.

## Conclusion

The current study results showed that non-compliance with therapeutic orders in outpatient settings depended on various factors that interacted with each other including individual-related factors, disease characteristics, therapy-related factors, health service provider features, and socioeconomic issues.

Therefore, health professionals and policymakers should be aware of such factors and consider them when designing programs to improve compliance with treatment. Education, adequate notification about diseases and their treatments can be useful to promote the individuals’ health literacy and knowledge. When doctors visit patients with low health literacy, they should speak slowly, repeat the information, use simple and non-medical language, and utilize learning techniques. Establishment of good communication and gaining patient’s trust, increasing patient participation in therapeutic decision-making, engaging the patient in designing his/her treatment plan, explaining accurately about his/her disease and treatment, and answering his/her questions can reduce non-compliance. In addition, considering the patient’s economic condition and therapeutic costs is one of the important factors that policymakers should pay attention to when they decide to improve the service provision system.

## Additional file

Additional file 1: The interview protocol for the semi-structured interviews. (DOCX 13 kb)

## Data Availability

The datasets analyzed during the current study are available from the corresponding author on reasonable request. Of-course, it should be noted that all interviews were conducted, transcribed and are accessible into Persian language.
