# Supplementary material for: Effective factors in non-compliance with therapeutic orders of specialists in outpatient clinics in Iran: a qualitative study
Source: BMC Health Serv Res. 2019 Jun 24;19:413. doi: 10.1186/s12913-019-4229-4 (PMC6591863; doi:10.1186/s12913-019-4229-4)
Supplement: Supplementary file 1 — The interview protocol for the semi-structured interviews. (DOCX 13 kb) [file 12913_2019_4229_MOESM1_ESM.docx]

**Additional file 1:** The interview protocol for the semi-structured interviews

**Interview guide**

1. What factors do you think may cause patients who go to outpatient clinics do not adhere/compliant medical/therapeutic orders prescribed by specialists? (e.g. medicines, surgery, non-invasive procedures, diet or life style, laboratory tests and diagnostic/medical imaging orders) (Please discuss about your experience)
2. What are the individual factors that patients who go to the outpatient clinics may not adhere/compliant their prescribed treatments? (Please discuss about your experience)
3. What are the factors associated with the provider (the specialist as a therapist and prescribing the treatment and or globally service delivery system) those may affect patients referred to clinics may not follow/adhere/compliant their treatments? (Please discuss about your experience)
4. In your opinion, what are the factors associated with socioeconomic issues that patients who come to outpatient clinics may not adhere/compliant their prescribed treatments? (Please discuss about your experience)
5. Are there other factors that you want to mention? (Please discuss about your experience).
6. On a basis of your earlier discussions, which factors are the most important challenges in non-compliance/non-adherence the patients of therapeutic orders at the outpatient clinics in Kerman? (Please discuss about your experience).
7. If you want to prioritize the mentioned factors, which factor/factors are your first priorities?
8. Is there anything else you would like to add in support of our study about the non-compliance/non-adherence in out-patient clinics in Kerman?
